# Metabolic survey of *Botryococcus braunii*: Impact of the physiological state on product formation

**DOI:** 10.1371/journal.pone.0198976

**Published:** 2018-06-07

**Authors:** Olga Blifernez-Klassen, Swapnil Chaudhari, Viktor Klassen, Robin Wördenweber, Tim Steffens, Dominik Cholewa, Karsten Niehaus, Jörn Kalinowski, Olaf Kruse

**Affiliations:** 1 Bielefeld University, Algae Biotechnology and Bioenergy, Faculty of Biology, Center for Biotechnology (CeBiTec), Universitätsstrasse 27, Bielefeld, Germany; 2 Bielefeld University, Proteome and Metabolome Research, Center for Biotechnology (CeBiTec), Universitätsstrasse 27, Bielefeld, Germany; 3 Bielefeld University, Fermentation Engineering, Faculty of Technology, Universitätsstrasse 25, Bielefeld, Germany; 4 Bielefeld University, Microbial Genomics and Biotechnology, Center for Biotechnology (CeBiTec), Sequenz 1, Bielefeld, Germany; Texas A&M University College Station, UNITED STATES

## Abstract

The microalga *Botryococcus braunii* is widely regarded as a potential renewable and sustainable source for industrial applications because of its capability to produce large amounts of metabolically expensive (exo-) polysaccharides and lipids, notably hydrocarbons. A comprehensive and systematic metabolic characterization of the *Botryococcus braunii* race A strain CCAP 807/2 was conducted within the present study, including the detailed analysis of growth-associated and physiological parameters. In addition, the intracellular metabolome was profiled for the first time and showed growth- and product-specific fluctuations in response to the different availability of medium resources during the cultivation course. Among the identified metabolites, a constant expression of raffinose was observed for the first time under standard conditions, which has until now only been described for higher plants. Overall, the multilayered analysis during the cultivation of strain CCAP 807/2 allowed the differentiation of four distinct physiological growth phases and revealed differences in the production profiles and content of liquid hydrocarbons and carbohydrates with up to 84% of organic dry weight (oDW). In the process, an enhanced production of carbohydrates with up to 63% of oDW (1.36±0.03 g L^-1^) could be observed during the late linear growth phase, whereas the highest accumulation of extracellular hydrocarbons with up to 24% of oDW (0.66±0.12 g L^-1^) occurred mainly during the stationary growth phase. Altogether, the knowledge obtained is potentially useful for the general understanding of the overall physiology of *Botryococcus braunii* and provide important insights into the growth behavior and product formation of this microalga, and is thus relevant for large scale biofuel production and industrial applications.

## Introduction

Renewable and sustainable energy sources from photosynthetic microalgae are gaining increasing attention in recent years [1,2]. Like all microalgae, *Botryococcus braunii* is capable of the conversion of sunlight and CO_2_ into biomass and valuable products. Because of its almost unique ability to synthesize large quantities of (exo-)polysaccharides and hydrocarbons and to secrete the latter [3–5], this colony forming green microalga represents a potential source for industrial applications like biofuels and other valuable chemicals [6,7].

The colonies of *B*. *braunii* feature a unique organization with the individual cells embedded in a hydrocarbon-containing extracellular matrix (ECM), which are encapsulated in a retaining cell wall surrounded by a fibrillary sheath layer [8]. Based on the chemical structure of the synthesized hydrocarbons, *B*. *braunii* is differentiated into four distinct races (race A, B, L and S) [3,4,9]. Race A strains of *B*. *braunii* produce mainly C_23-_C_33_ odd numbered *n*-alkadienes and *n*-alkatrienes [10], derived from fatty acid via elongation-decarboxylation pathway [3,11,12]. Races B and L produce isoprenoid-derived hydrocarbons: methylsqualenes and C_30_–C_37_ botryococcene triterpenoids in race B [13] and the C_40_ tetraterpenoid lycopadiene in race L [14]. Recently tentatively identified race S strains synthesize C_18_ epoxy-*n*-alkanes and C_20_ saturated *n*-alkanes [15]. The synthesized hydrocarbons accumulate likely for storage in the extracellular space, in contrast to other examined microalgae, which store lipid bodies in the cytoplasm [16–18]. In addition, *B*. *braunii* is also well known to synthesize large amounts of polysaccharides, which represent the components of the cell wall and the retaining wall/ fibrillar sheath [8,18]. The polysaccharide precursor are supposedly synthesized in the Golgi body and were proposed to be delivered via Golgi vesicles through the fenestrated ER, across the cell membrane/wall to the retaining wall and its associated fibrillar sheath [8]. During the growth of *B*. *braunii* cultures, uniform cup-shaped structures, also called “shells”, which represent excised segments of the retaining wall and its fibril sheath, are accumulating in the surrounding media and causing viscosity increase of the culture [3,8,19].

However, despite the remarkable capability to synthesize and excrete industrially interesting chemicals into the extracellular matrix, the comparably slow growth rates of *B*. *braunii*, most likely associated with the production of high energy compounds like hydrocarbons and other polymers, challenge the marketable application of this microalga [9,20]. Many previous studies, dedicated to the determination of optimal culture conditions have clearly indicated that factors like culturing conditions and nutrient availability have drastic effects on growth and productivity performance of *B*.*braunii* [3–5,9]. Generally, oil-accumulating microalgae produce fatty acids or triglycerides under environmental stresses such as nutrient depletion [21], but the highest hydrocarbon productivity in *B*. *braunii* has been reported to appear during the active growth phase [11,13,17,20]. On the other hand, Dayananda and co-workers demonstrated significant differences of the growth-associated hydrocarbon production under varying culture media and conditions [22]. The production of polysaccharides has been described to occur during linear and stationary growth phases with enhanced production rates during algae growth decline phase, probably due to the development of nitrogen limitation in the culture medium [3,23]. But above all, the performed studies have pointed out the general occurrence of huge variations in productivity (e.g. hydrocarbons and polysaccharides) and growth behavior across different races and species of *B*. *braunii* [24–26], observable even for the same stain [22,27]. For instance, *Botryococcus braunii* race A strain CCAP 807/2 was previously assessed to be of potential interest for biofuel production, because of its relatively high extractable hydrocarbon content with over 30% of dry weight (DW) in comparison to other race A strains [26,28]. On the other hand, another recent study reported for the same strain quite contradictory results, showing either a high biomass productivity with only low hydrocarbon and polysaccharide concentrations (7% and 5% of DW, respectively) in bubble column cultivation, or *vice versa* in shake flasks [27]. Moreover, Moutel and co-workers reported an even totally diminished growth with maximal reached biomass concentrations of 0.01g L^-1^ for *B*. *braunii* CCAP807/2 [29]. In this context, it is becoming obvious, that the application of different cultivation conditions and continuance, but also apparently the physiological state of the cells, have a tremendous impact on the production rates of biomass and of the product of interest.

Although, a plethora of extensive work has been performed in the past, the physiological behavior of *Botryococcus braunii* cells is still not fully understood, since focus has been mainly set on fatty acids, hydrocarbons and carbohydrates. However, an upstream investigation of metabolic pathways may reveal more insight into their regulation and provide valuable knowledge for future reasonable strain selection and optimal productivity. Metabolomics represents an important component of system biology research that can be readily used to study phenotype differences through the analysis of intra- and/or extracellular metabolites. It is a sensitive tool for the evaluation of the responses of organisms to various interferences and is considered as the endpoint of all biological processes, and thus carries the influence of all genetic and environmental factors [30].

Therefore, due to the discrepancy of the previous observations and limited information on the intracellular metabolome, the present study aimed to perform a comprehensive and systematic metabolic characterization of the *Botryococcus braunii* race A strain CCAP 807/2 to elucidate the impact of the physiological state of the cells on product formation. Besides the detailed analysis of growth-associated and physiological parameters, the intracellular metabolite fluctuations were profiled via GC-MS for the first time in response to the different availability of medium resources during the course of the cultivation of 30 days. The acquired data allowed the differentiation into different growth stages based on nutrient availability in the culture and illustrated the obvious correlation between cell physiology and productivity capacities of *B*. *braunii* cells. Therefore, the knowledge obtained within this work will be useful for an adequate evaluation of the future strains of interest, in order to be able to assess the growth behavior and the productivity characteristics thereof.

## Material and methods

### 2.1. Strain and culture conditions

Liquid, non-axenic cultures of *Botryococcus braunii* CCAP 807/2 (Grasmere, Cumbria, England) were phototrophically cultivated at 25–28°C under 16:8 light-dark illumination of 350–400 μmol photons m^-2^ s^-1^ white light and continuous agitation in modified Chu-13 medium [18] (pH of 7.2) with the following composition: 734.0 μM CaCl_2_ × 2H_2_O; 811 μM MgSO_4_ × 7H_2_O; 602 μM K_2_HPO_4_; 3.95 mM KNO_3_; 50 nM Na_2_O_4_Se; 50 μM FeNaEDTA; 0.32 μM CuSO_4_ × 5H_2_O; 0.76 μM ZnSO_4_ × 7H_2_O; 0.32 μM CoSO_4_ × 7 H_2_O; 7.94 μM MnSO_4_ × 4H_2_O; 0.25 μM Na_2_MoO_4_ × 2H_2_O; 43.26 μM H_3_BO_3_; 1mL 99.99% (v/v) H_2_SO_4_. Carbon supply was achieved by bubbling the cultures with moisture pre-saturated carbon dioxide-enriched air (6%, v/v) with a flow rate of 10 L h^-1^. The cultivation was performed in cylindrical glass bottles (with maximal capacity of 3.3 L) with a total starting volume of 2.5 L per replicate. pH measurements of the cultures were taken periodically (pH-Meter Qph 70, VWR, Darmstadt), to ensure that the high CO_2_ input did not result in a lower pH and affect the physiological culture condition (S1 Fig). Culture growth was monitored by measurements of organic dry biomass weight (oDW) and chlorophyll concentration.

### 2.2. Determination of growth parameters

The biomass concentration was determined either by obtaining a pellet of 10 mL of cell culture (3000 × *g* for 5 min) or using 10 mL whole culture (in at least three technical replicates per sample) and drying of the cell pellet/culture in a pre-weighed glass tube at 105°C for 24 h. To determine the organic biomass fraction, the sample tubes were subsequently incubated at 550°C following the protocol according to Astals *et al*. [31] and the residual ash content was determined by weighing. The amount of organic biomass (dry weight minus the ash content) was calculated and expressed as organic dry weight of cells and whole culture biomass (cell oDW and culture oDW), respectively. The total protein concentration of the whole culture was determined by Lowry assay (Bio-Rad, CA, USA), according to manufacturer’s instructions. The chlorophyll concentration was obtained from cell pellets of 1 mL culture samples, centrifuged at 10000 × *g* for 5 min. Chlorophyll molecules were extracted by addition of 1 mL methanol followed by incubation for 30 min at 60°C in the dark. The insoluble matter was removed by centrifugation at 16,100 × *g* for 1 min. The absorbance at 666, 653 and 470 nm were recorded with a spectrophotometer (Thermo Scientific, Genesys 10s UV-vis) and total chlorophyll as well as total carotenoids content was calculated according to Wellburn *et al*. [32].

### 2.3. Microscopic analysis

Microalgae cells were regularly monitored by optical microscopy (Motic BA310, Motic, China). For visualization of lipids and hydrocarbons, 500 μL *B*. *braunii* culture were treated with 4 μL of 100 mg L^-1^ BODIPY 505/515 stain (4,4-difluro-1,3,5,7-tetramethyl-4-bora-3a,4a-diaza-s-indacene, Thermo Fischer Scientific), prepared in 40% (v/v) DMSO), with addition of 0.1% (v/v) of TritonX, followed by incubation in the dark for 10 min at room temperature. Fluorescence microscopy was performed with an epifluorescence microscope at 63x magnification (DM600B Leica microsystems, Wetzlar, Germany).

### 2.4. Measurement of elemental C and N content in the biomass (C/N ratio)

Total carbon (C) and nitrogen (N) content of the algal cell and whole culture biomass (2–3 mg lyophilized biomass per sample; measured in three technical replicates) was determined via an element analyzer (VARIO EL III, Element Analyzer, Hanau, Germany). Helium was used as carrier gas and thermal conductivity detector (TCD) for gas (N_2_, CO_2_ and H_2_O) quantification. BBOT (2.5-Bis(5-tert-butyl-benzoxazol-2-yl)thiophene) was used for the verification of the instrument calibration and standards. All parameters were applied according to the manufacturer specifications.

### 2.5. Measurement of nitrogen content in the culture media

Nitrate nitrogen (NO_3_ –N) content in the cell free supernatant was analyzed using a standardized Hach Lange nitrate cuvette test (LCK339) and measured in a DR 3900 Spectrophotometer (HachLange, Germany) according to the manufacturer’s instructions.

### 2.6.Extraction and analysis of metabolites and total lipids

For the determination of metabolites and total lipids, algal biomass was harvested by centrifugation (16000 × *g* for 1 min) at three different time points (6, 15 and 30 days) during the cultivation, immediately frozen in liquid N_2_, lyophilized and then stored at -80°C.

The extraction, derivatization and measurement of metabolites were performed as described previously [33]. In brief, 10 mg of freeze-dried algal cell biomass was mixed with 1 mL of 80% methanol (v/v) containing 10 μM ribitol (internal standard) and 300 mg of silica beads of 0.1 mm diameter (Carl Roth, Karlsruhe, Germany) and disrupted using Precellys homogenizer (three times at 6,500 rpm for 45 s, Peqlab, Erlangen, Germany). The mixture was centrifuged at 21,000 × *g* for 20 min and the supernatant was treated, after drying under N_2_-stream, with 100 μL of methoxyl-amine hydrochloride (Sigma-Aldrich, Steinheim, Germany) in pyridine (20 mg/ mL^-1^) for 90 min at 37°C while stirring. After the incubation, 30 μL of N-Methyl-N-(trimethylsilyl- lyl) trifluoroacetamide (Macherey & Nagel, Düren, Germany) were added, and the mixture was further incubated for 30 min at 37°C with constant stirring and subsequently analyzed via GC-MS. Relative abundance of primary metabolites was expressed as the percentage of all the identified metabolites after normalization to the internal standard ribitol. The unidentified peaks, accounting for only a small fraction of the total detected relative peak area (10–20%, S2 Fig), were not included in the evaluation.

The extraction of total lipids and the evaluation of the fatty acids, derived from the polar and non-polar lipid fraction was carried out according to Bogen *et al* [34], using a modified Folch method [35]. Briefly, total lipids were extracted from 50 mg homogenized dry cell biomass (30 s at 6,500 rpm, Precellys 24, Peqlab, Erlangen, Germany) with 8 mL chloroform and 4 mL methanol (ratio 2:1 (v/v) for CHCl_3_ and CH_3_OH), followed by subsequent washing step with 3 mL H_2_O. The organic phase, containing total lipids was separated in a new pre-weighed glass vial and dried in N_2_ atmosphere. The extracted total lipid content was determined gravimetrically. Then the total lipids were separated into two fractions as polar and non-polar lipids by column chromatography using silica gel 60 (70–230 mesh, Merck, Darmstadt, Germany), according to the protocol of Li *et al*: 6 volumes of chloroform or methanol were used to collect the polar and non-polar lipids, respectively [36]. The content of each lipid fraction was also determined gravimetrically and then derivatized to gain fatty acid methyl esters (FAME). FAME were obtained by incubation of the polar and non-polar lipid fractions (P lipid and N-P lipid, respectively) for 2 h at 80°C with methanol, hydrochloric acid and chloroform (10:1:1 v/v), followed by extraction using a solution of hexane and chloroform (4:1 v/v) and analysed via GC-MS. For the determination of the relative amount of fatty acids, C_17_-triacylglycerol (50 μg, glyceryl triheptadecanoate, Sigma-Aldrich, Steinheim, Germany) was added to each sample as an internal standard. The peaks were identified by comparison of retention times and mass spectra to a Supelco 37 component FAME mix (Sigma-Aldrich). Total ion chromatograms were used to calculate the relative abundance of fatty acids and hydrocarbons after normalization to the internal standard.

### 2.7. GC-MS

GC-MS analysis was performed with a TraceGC gas chromatograph and a ITQ ion trap mass spectrometer equipped with an AS2000 auto sampler (ThermoFinnigan, Dreieich, Germany) according to a previous study [37]. In brief, sample volume of 1 μL was splitlessly injected at 300°C injector temperature. The gas chromatograph was equipped with a 30-m × 0.25-mm VF-5 ms column having a 0.25 μm 5% diphenyl and 95% dimethylsiloxane coating (Varian Deutschland GmbH, Darmstadt, Germany). The interface temperature was adjusted to 250°C and the ion source was set to 200°C. Helium carrier gas was set to a constant flow of 1 mL min^-1^. Following 1 min of constant heating at 80°C, the oven temperature was raised gradually by 6°C min^-1^ to 300°C where it was held for 5 min. Mass spectra were recorded at 20 scans s^-1^ with a scanning range of 50–750 m/z.

The evaluation of the chromatograms was accomplished with the Xcalibur software (ThermoFinnigan). Respective metabolites were identified by comparison with the NIST 05 library (National Institute of Standards and Technology, Gaithersburg, MD; ThermoFinnigan), the Golm Metabolome Data base (MPI, Golm, Germany), and verified with purified standards (Sigma-Aldrich). All spectra were manually reviewed and quantified based on the internal standards.

### 2.8. Pigment analysis via UV-vis-HPLC

For intracellular pigment analysis, 3–5 mg of lyophilized dry biomass was mixed with 0.1 mm silica beads and 1 mL of 90% acetone (v/v, analytical grade saturated with CaCO_3_) and homogenized (three times at 6,500 rpm for 45 s, Precellys 24, Peqlab, Erlangen, Germany). The particle-free supernatant was obtained after centrifugation at room temperature (5 min, 16,000 × *g*). Pigments were separated via UV-HPLC (Thermo Finnigan) as described before [38]. Briefly, 20 μL of pigment sample was separated on a AccucoreTM Polar Premium RP-C18 column (150 mm × 4,.6 mm, 2.6 μm particle size, Thermo Scientific) with a pre-column Accucore C8 filter (10 × 4.6 mm, 2.6 μm). For separation, eluent A with 0.1 M ammonium acetate/methanol (15:85, v/v), and eluent B with methanol/acetonitrile/acetone (44:43:13, v/v) were used. The column was equilibrated with a flow rate of 0.5 mL min^-1^ for 5 min with 100% eluent A. The solvent composition changed at 31 min to 75% A and 25% B, and then to 100% B at 47 min until the end of run at 70 min. The UV-vis-detector scanned the wavelength range of 190–800 nm with a bandwidth of 1 nm and a scan rate of 1 Hz, and additionally, a discrete channel at 440 nm was recorded. The resulting chromatograms were evaluated with the Xcalibur software (Version 2.0.7, Thermo Scientific). Pigments were identified based on commercially available pigment standards (DHI group), and normalized to the applied dry biomass content. The percentage of relative abundance was obtained from the peak area per mg of dry biomass, and the total peak areas of all pigments detected at Phase II was considered as 100%.

### 2.9. External hydrocarbon extraction and analysis via GC-MS and GC-FID

Hydrocarbon extraction was performed according to Khatri *et al* with additional heating step at 85°C in order to increase the recovery of hydrocarbons [39,40]. In brief, 1.5 to 3.0 mL of culture was mixed with equal volume of acetone and *n*-hexane and vortexed for 2–3 min. After a heating step of 5 min at 85°C in water bath, the extract was centrifuged at 3020 × g for 5 min and upper *n*-hexane phase was collected. The extracted hydrocarbon-containing *n*-hexane phase was dried in a N_2_-atmosphere and resuspended in 500 μL of *n*-hexane, containing an internal standard *n*-hexatriacontane (C_36_H_74_) and analysed via GC-MS as described above. Additionally, samples were analyzed in a Shimadzu GC-2010 Plus (Shimadzu, Kyoto, Japan) unit with a FID detector as described previously [41]. Briefly, the separation was performed with a Macherey-Nagel OPTIMA FFAPplus column (30 m×0.25 mm ID, 0.40 mm OD and 0.50 μm film) and a 10 m pre-column (carbowax-deactivated, 0.25 mm ID, 0.40 mm OD, Macherey-Nagel). The sample injection volume was 1 μL working in split mode (1:5) and injected with an auto-injector (Shimadzu AOC-20i). The analysis was conducted in a constant pressure-mode of with 160 kPa with helium as carrier gas and nitrogen as makeup gas (Linde, München, Germany). The method consisted of a temperature ramp from 55 to 232°C at 6°C min^-1^ and to 250°C at 3°C min^-1^, with keeping the final temperature of 250°C constant for 29.5 min. For identification and quantification of all hydrocarbons external standards (alkane standard solution C_21_-C_40_, Sigma-Aldrich/Fluka) were used.

### 2.10. Analysis of total carbohydrates and evaluation of the sugar monomers

The amount of total carbohydrates of algal fresh cultures and cell-free supernatant was determined in micro plates, using a modified DuBois-assay [42]. In brief, 100 μL of conc. H_2_SO_4_ were mixed with 30μL of ice-cooled samples, followed by the addition of 20 μL of aqueous 5% phenol solution. The plate was incubated at 100°C for 15 min and subsequently cooled on ice for 5 min. After an additional incubation of 10 min at room temperature, the absorbance was measured at 490 nm and quantified based on glucose standard.

In addition, for the GC-MS analysis the supernatant containing carbohydrates were derivatized into sugar monomers by methanolysis and peracetylation according to Steffens *et al* and analysed via GC-MS [43]. Briefly, 100 μg of freeze dried culture supernatant were used for methanolysis in 0.5 M HCl / MeOH for 45 min at 85°C. After washing with methanol and drying under nitrogen flow, samples were peracetylated using pyridine:acetic anhydride (2:1 v/v) for 30 min at 85°C. After the peracetylation step, the samples were washed with chloroform and resuspended in 100 μL of chloroform and analyzed via GC-MS. In addition, in order to confirm the presence of galactose in the culture supernatant, the samples were spiked with glucose or galactose prior the GC-MS measurement.

### 2.11. Statistical analysis

Statistical analysis was performed with two-tailed Student’s t-test, resulting in p-values indicated by asterisks (p≤0.05 = *, p≤0.01 = **). Results were shown either as mean value or fold-change of mean values; error bars represent standard deviation or standard error of mean (SD/SEM, n = 3).

## Results and discussion

### 3.1. Evaluation of distinct growth phases

**Fig 1:**
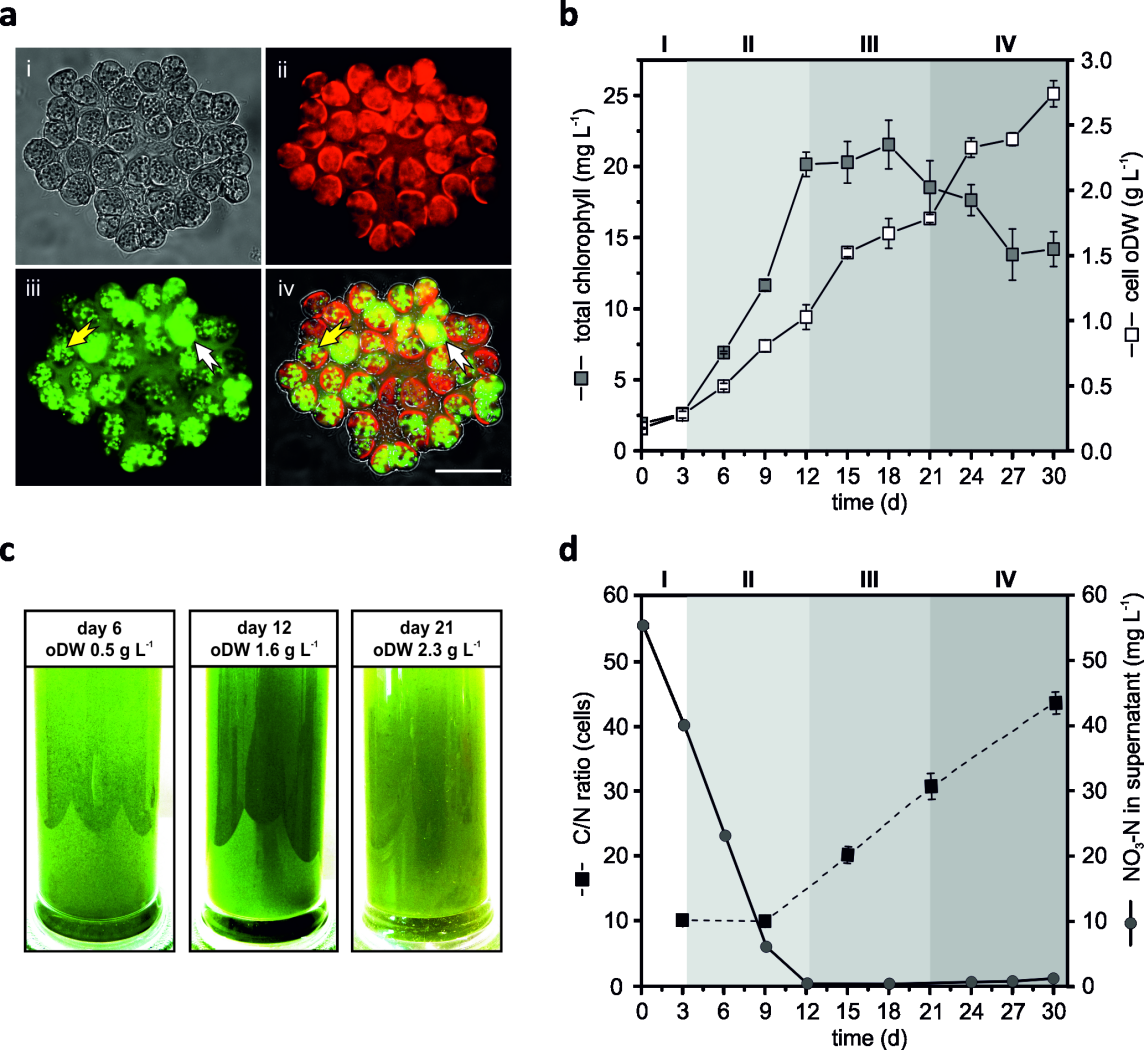
Growth performance analysis of *Botryococcus braunii* CCAP 807/2 and differentiation into different growth stages. **a.** BODIPY505/515 staining of cell colonies after 12 days of cultivation. (i) DIC image of single BODIPY505/515-stained colony, (ii) chlorophyll autofluorescence channel, (iii) BODIPY channel, false colored green, (iv) Merge of DIC, BODIPY, chlorophyll autofluorescence images. Bar 25 μm. Yellow and white arrows indicate cytoplasmic oil bodies and extracellular hydrocarbons, respectively. **b.** Determination of total chlorophyll and organic dry weight (oDW) of the cell biomass during cultivation period of 30 days. **c.**Illustration of the color change of the cultures during the course of the cultivation. Images show the cultures after 6, 12 and 21 days of culturing as well as the corresponding biomass dry weight. **d.** Measurement of the C/N ratio and nitrogen (NO_3_-N) content in the media supernatant during cultivation period of 30 days. Numbers at the top of the graph (I to IV) represent the proposed distinct growth stages of *B*. *braunii* cells, referred as Phases I–IV. Error bars represent standard error of mean value of three biological and three technical replicates (SE; n = 9).

The colony forming green microalga *Botryococcus braunii* race A strain CCAP807/2, in focus of this study, is capable of synthesizing hydrocarbons and (exo-)polysaccharides [27] and accumulating long-chain hydrocarbons as oil droplets in the cytoplasm and in the extracellular matrix (Fig 1A).

The growth performance of *B*. *braunii* CCAP 807/2, analyzed by measuring the organic dry weight (oDW) of the cell biomass, showed a continuous increase in the time course of the cultivation of 30 days, yielding 2.74±0.1 g L^-1^ with maximum biomass productivity of up to 104 mg L^-1^ d^-1^ (Fig 1B). Nevertheless, despite the constant biomass accumulation, obvious physiological changes could be observed during the cultivation period, i.e. a change of the culture color from green to pale yellowish-green (Fig 1C), strongly indicating different physiological growth phases of the cells [3]. To retrace the different growth phases of CCAP 807/2, the total chlorophyll content was used as a marker for the cell proliferation since the conventional cell count method was not suitable for this colony forming alga. The determined total chlorophyll content revealed, except for the initial period of the first three days (considered as Phase I), a gradual increase of up to 20.15±0.86 mg L^-1^ on day 12, thereby indicating a linear growth phase (Phase II). The chlorophyll concentration stagnated after day 12, suggesting the onset of the stationary phase (Phase III); and finally declined steadily from day 18 to 14.19±1.22 mg L^-1^ (Phase IV, Fig 1B). Additionally, this assumption was further supported by the measurements of the total protein concentration of the whole culture broth, showing a strong deceleration of the overall content after day 9 of the cultivation (S1 Fig). Since microalgae biomass usually contains high amounts of proteins [44], which in turn have incorporated nitrogen, the ratio of carbon to nitrogen (C/N ratio) was determined as an indicator for the overall presence of nitrogen in the cells. The measurement of total elemental carbon and nitrogen content in *B*. *braunii* CCAP 807/2 cell biomass revealed that the C/N ratio remained constant with a value of up to 10 between days 3–9 (Fig 1D), indicating that the cells did not suffer any nitrogen limitation [45]. The further observed gradual increase of the C/N ratio from 10.0±0.2 (day 9) to the value of 43.9±1.9 at the end of the cultivation (Fig 1D), implies progressive nitrogen limitation. Additionally, the concentration measurements of the nitrogen source (KNO_3_) in the culture supernatant during the cultivation revealed that the major portion of nitrogen in the culture media was absorbed by the cells already after 9 days (Fig 1D). Nutrient limitation, occurring in consequence of resource depletion in the culture medium during the cultivation, is well known as a trigger for the reduction in amino acid synthesis activity, and consequently also lowers the *de novo* synthesis activity of new proteins. Under these conditions, algal cells tend to accumulate more carbon-rich storage compounds as polysaccharides (e. g. starch) and/or lipids [21,46], which in turn is reflected in the increasing C/N ratio. It is worth mentioning, that the observed C/N ratio values within this study are much higher compared to earlier reported values of 27 for *B*. *braunii* strain Showa [47] and other microalgae with values of 24–26 under nitrogen depletion [45]. Therefore, these findings suggest, that the increase of the culture biomass after days 9–12 was mainly due to the accumulation of carbon-rich metabolites such as sugars and/or lipids (in particular hydrocarbons) instead of proteins.

Collectively, based on the results presented above, four distinct stages of growth can be envisaged for the presented batch cultivation of *B*. *braunii* race A CCAP807/2 (Phases I–IV, indicated by the shades of gray in the Fig 1B).

### 3.2. Intracellular metabolic response during proposed growth stages

**Fig 2:**
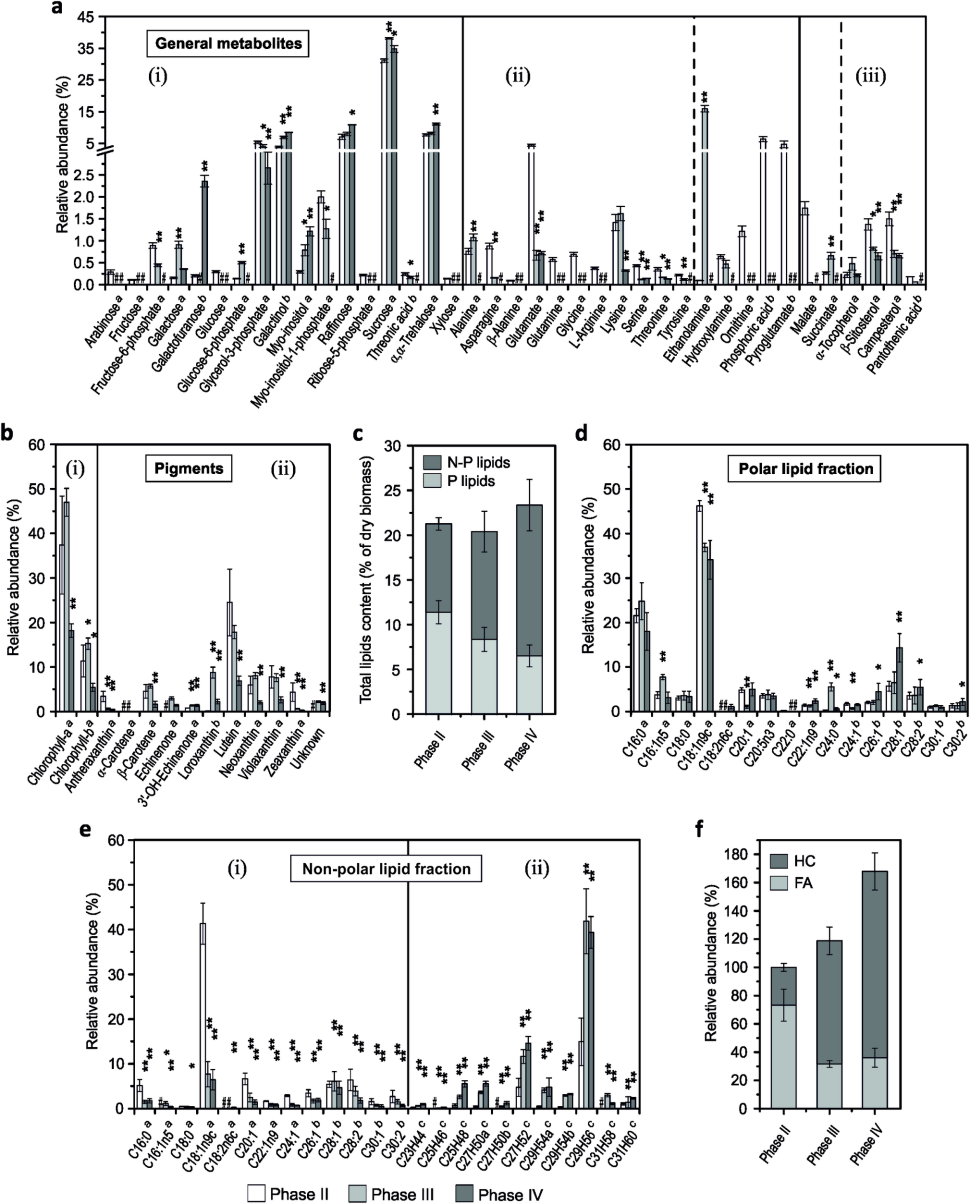
The overall intracellular metabolome profile of *Botryococcus braunii* CCAP 807/2, containing all identified metabolites during the proposed growth stages, referred as Phases II (linear phase), III (stationary phase) and IV (decline phase). **a.** Non-targeted metabolome profile of primary metabolites showing the comparison of relative abundance level of metabolites, divided into three different categories based on the related metabolic pathways, thus (i) glycolysis intermediates, sugars and sugar alcohols, (ii) amino acids and other related metabolites and (iii) citric acid cycle intermediates, terpenoids, steroids and vitamins. **b.** Intracellular pigments with relative abundances of (i) chlorophylls and (ii) carotenoids. **c.** Gravimetrically determined total lipid content, containing polar (P lipids) and non-polar lipid (N-P lipids) and expressed as percentage of dry biomass weight. **d.** Polar lipid fraction with relative abundance level of fatty acids. **e.** Non-polar lipid fraction with relative abundance levels of (i) fatty acid and (ii) hydrocarbons. **f.** Comparison of hydrocarbons and fatty acids derived from the total non-polar lipid fraction on the basis of the relative abundance levels, considering Phase II as 100%. Metabolites were identified by ‘*a*’ comparison with the NIST 05 library and Golm Metabolome Database (Lib) and verified with purified standards; ‘*b*’ only via above mentioned databases with RSI value above 750. ‘*c*’ marks the identified hydrocarbons via mass spectra of GC-MS and available literature [10,17]; ‘#’ not detected. Error bars represent standard deviation (SD). Asterisks represent *p-values* as determined via Student’s t-test (* = < 0.05, ** = < 0.01).

In order to gain insights into the cellular metabolic response of *B*. *braunii* CCAP807/2, a detailed analysis of samples after 6, 15 and 30 days of cultivation (Phase II, Phase III and Phase IV, respectively) was performed, aiming the determination of the overall intracellular metabolome profile and subsequent changes during the proposed distinct growth phases. These analyses included the determination of non-targeted primary metabolites, fatty acids derived from polar and non-polar lipid fractions as well as the profiling of photosynthetic pigments involved in photo protective mechanisms (Fig 2, S1 Table).

Among the identified metabolites (Fig 2A, S1 Table), sugars such as sucrose, raffinose, and α-α-trehalose appeared to be the most abundant and increased slightly towards the end of cultivation (Phases III and IV) in comparison to the linear phase (Phase II) (Fig 2A, i). Of the sugar-related metabolites, sucrose turned out to be the most abundant component (31.1±0.6%) followed by some other sugar compounds such as α-α-trehalose (7.7±0.4%) and raffinose (7.0±0.8%) within the linear growth phase (Phase II). In higher plants, sucrose (α-D-glucopyranosyl-β-D-fructofuranoside), commonly known as the prime product of photosynthesis, represents the main form of translocated carbon and the main substrate for sink metabolism [48]. Furthermore, sucrose has been reported to possess significant regulatory and integrative functions, and its metabolism is closely linked to the metabolism of inorganic and organic nitrogen, and thus may be part of a broader mechanism for balancing resource acquisition and allocation [48,49]. The disaccharide trehalose (α-D-glucopyranosyl-α-D-glucopyranoside) is, beside its main task as a storage molecule, strongly enhanced under stress conditions, playing a role as a stress-responsive factor with a bio-protective role, helping the cells to retain cellular integrity and stability [50]. The galactotrisaccharide raffinose is synthesized from sucrose by the subsequent addition of a activated galactose moiety donated by galactinol (1-*O*-α-D-galactopyranosyl-L-*myo*-inositol), which is formed by the galactinol synthase using uridine diphosphate galactose and *myo*-inositol as substrates [51]. Interestingly, the relative levels of the metabolite *myo*-inositol were found to be increased up to 2.7 and 4.1-fold during the Phases III and IV when compared to the levels in the linear phase (Phase II) (Fig 2A, i; S1 Table). This increase is in good agreement with the observed high levels of raffinose and galactinol (Fig 2A, i), since the reduction of *myo*-inositol content was reported to result in strongly reduced levels of galactinol and raffinose [52]. The synthesis and accumulation of raffinose and other higher molecular weight compounds from the raffinose family of oligosaccharides (RFOs) has been so far exclusively reported for higher plants [51], with the only exception of a cold-stress-induced raffinose accumulation in the green alga *Chlorella vulgaris* [53].

In contrast to di- and oligosaccharides, monosaccharides such as glucose, arabinose, fructose and xylose were only detected during the linear growth (Phase II), except for galactose, which was found to be increased up to 5.9 and 2.3-fold, respectively, during the Phases III and IV. Another exception is galactofuranose, the five-membered ring form of galactose, which increased up to 11.5-fold during the Phase IV compared to the linear growth phase (Fig 2A, i; S1 Table). Galactofuranose has been reported to be crucial for the elaboration of the cell surface coat, since its absence often results in morphological abnormalities and an impaired cell wall function [54]. Thus, the elevated levels of galactofuranose at the end of the cultivation could be due to increased incorporation of these components into the *B*. *braunii* cell walls in order to maintain and stabilize them.

Furthermore, the identified intermediates (except glucose-6-phosphate and succinate) of the glycolysis, the pentose phosphate pathway and the citric acid cycle showed a steady decrease towards the end of the cultivation (Fig 2A). An up to 2.5-fold increase of succinate during the Phase III compared to Phase II (Fig 2A, iii; S1 Table) might serve as an indication for the up-regulated glyoxylate cycle, which provides succinate for further conversion into longer-carbon-chain compounds via gluconeogenesis [55]. The observed accumulation of glucose-6-phosphate (G6P) (up to 3.7-fold during Phase III in comparison to Phase II (Fig 2A, i; S1 Table)), is also likely to provide NADPH as a reduction equivalent for fatty acid synthesis via its oxidation in the pentose phosphate pathway [56]. Moreover, the conversion of G6P to *myo*-inositol-1-phosphate represents the first committed step in the *myo*-inositol biosynthesis, a biochemical reaction involved in many aspects of organism physiology such as sugar storage and transport, carbohydrate metabolism, cell wall formation as well as stress physiology [57]. Overall, the high abundancy of the sugar metabolites observed during the cultivation strongly suggests that *B*. *braunii* CCAP807/2 produces large amounts of carbohydrates at all stages of growth, however with different consumptions and dedicated to different functions within the different growth phases.

In addition, the conducted metabolic analysis demonstrated a significant decrease in the abundance of various amino acids and other related compounds (Fig 2A, ii). All identified metabolites of this specific class except for alanine, lysine and ethanolamine, decreased or could not be detected at all during the Phases III and IV in comparison to the linear growth phase (Phase II). Especially, the decrease (or absence) of proteinogenic N-rich amino acids such as glutamate, glutamine, asparagine, and arginine during the Phases III and IV might serve as a further indication for nitrogen insufficiency of *B*. *braunii* cells in the later stages of the cultivation, as suggested above. In fact, glutamate and glutamine are well known to represent the central metabolites in the nitrogen metabolism of plants and mammalia [58,59]. A low inorganic nitrogen content in the exponential growth phase typically results in a depletion of glutamine (the first amino acid formed during ammonium assimilation) as well as in decreased levels of many other amino acids, and consequently in decreased levels of proteins and other N-containing structural components like chlorophylls [60,61]. Interestingly, the relative abundance level of ethanolamine increased drastically up to 168-fold from Phase II to Phase III and could not be detected at the end of the cultivation (Fig 2A, ii; S1 Table). Ethanolamine is well known to be essential for the synthesis of phosphatidylethanolamine (PE) and phosphatidylcholine (PC), which represent the two major phospholipids in eukaryotic cells [62]. Furthermore, it was previously suggested to represent a potential marker for improving lipid accumulation since it is indirectly involved in fatty acid biosynthesis by stimulating the formation of oleic acid [63].

Moreover, within the metabolome analysis campesterol and ß-sitosterol were identified to represent the main sterols produced by *B*. *braunii* CCAP 807/2 strain. This finding is in good agreement with previous reports for the three *Botryococcus braunii* races (A, B and L), synthesizing in addition to ß-sitosterol, also campesterol or stigmasterol and to a lesser extent cholesterol [14,64]. Although the biosynthetic pathways of the predominant membrane sterol precursors are similar in *B*. *braunii* and *C*. *reinhardtii*, as suggested by the transcriptome analysis of race B Showa [65], the type of sterols synthesized by *B*. *braunii* resemble rather those of the higher plants than those known from green algae [66,67]. In higher plants, sitosterol and campesterol were reported to be able to regulate the membrane fluidity and permeability, and stigmasterol is specifically required for cell proliferation [66]. The active synthesis of sterols has been reported to occur during rapid cell division with concomitant synthesis of new membranes and to reduce drastically under low nitrogen conditions [68]. Both, sitosterol and campesterol were found to be decreased in the later stages of growth (Phase III and IV) to approximately up to 2-fold in comparison to Phase II (Fig 2A, iii), thus, indicating an overall decreased cell proliferation.

Apart from the metabolites mentioned above, intracellular photosynthetic pigments of *B*. *braunii* CCAP 807/2, involved in light harvesting and photo-protective mechanisms, were also analyzed and found to be composed of chlorophyll a, chlorophyll b as well as the carotenoids antheraxanthin, neoxanthin, loroxanthin, violaxanthin, zeaxanthin, lutein, α and β-carotene, echinenone and 3’-hydroxyechinenone (Fig 2B, S1 Table). All photosynthetic pigments were found to be increased during Phase III as compared to Phase II except for antheraxanthin, lutein, and zeaxanthin. In contrast, most pigments were significantly reduced (except for echinenone, 3’-hydroxyechinenone and an unknown carotenoid) at the end of the cultivation during Phase IV. Interestingly, the non-polar keto-carotenoid echinenone could not be detected in Phase II and only appeared during later stages of the cultivation (Fig 2B, Phase III and IV). In previous research, the initiation of echinenone synthesis was suggested to be concomitant with the onset of nitrogen deficiency in the culture medium [69], since this secondary carotenoid is produced during the stationary phase and then further transported to the lipid-rich matrix, where it is most likely involved in light protective mechanisms [70]. Moreover, it has been proposed that the color change of the *B*. *braunii* culture (Fig 1C) is mainly attributed to the accumulation of echinenone in the intracellular matrix and a concomitant decrease in intracellular pigments [71]. Additionally, apart from the chlorophylls a and b, the carotenoid lutein was found to be the most abundant pigment at phase II (24.6±7.5%), which is in good agreement with previous reports for lutein being the dominant carotenoid in *B*. *braunii* in the linear phase of growth [69,70]. However, the relative level of lutein decreased during further cultivation up to 1.4 and 3.6-fold (to 17.8±1.5% and 6.9±1.0%) in phases III and IV in comparison to the linear growth phase (Fig 2B; S1 Table), thus underlining once again the onset of the stationary phase as suggested before [3,69,70].

In addition, within the examination of the intracellular metabolic response of the proposed growth phases (*viz* Phases II, III and IV at cultivation days 6, 15 and 30, respectively) of *B*. *braunii* CCAP807/2, the analysis of total intracellular lipid content and subsequent evaluation of the fatty acid profile was conducted (Fig 2C, 2D and 2E; S1 Table). The gravimetrically determined total lipid content reached 21.2±1.9% and 20.4±3.1% of dry biomass weight (DW) at Phases II and III, respectively, followed by a further slight increase to 23.3±1.4% of DW at the end of the cultivation (Phase IV) (Fig 2C). Although, the observed values are comparable to other *B*. *braunii* race A strains [26,29], the overall modest increase in total lipid content favors the assumption that *B*. *braunii* CCAP807/2 might rather prefer carbohydrates for storage, instead of lipids, similar to the green alga *Chlamydomonas reinhardtii* [36]. While the total polar lipid content successively decreased with the progressing cultivation (from 11.4±1.2% to 6.5±1.2% of DW at Phase II and IV), the non-polar lipid content elevated from 9.8±0.6% of DW at Phase II to 16.8±2.8% of DW at Phase IV (Fig 2C).

Detailed analysis of the fatty acid profiles derived from polar and non-polar lipid fractions of *B*. *braunii* CCAP 807/2 revealed a diverse composition (Fig 2D and 2E). For fatty acids derived from the polar fraction, palmitic (C_16:0_), oleic (C_18:1n9c_) and octacosenoic (C_28:1_) acids were found to be the most abundant in all analyzed growth stages i.e. Phase II–IV, representing up to 74% of all identified fatty acids in this fraction (Fig 2D, S1 Table). This finding is in good agreement with earlier observations for the three races (A, B and L) of *B*. *braunii* [14]. The relative abundance of oleic acid decreased within the polar lipid fraction in the time course of the cultivation from 46.2±1.2% (Phase II) to 34.1±4.3% (Phase IV) and even more within the non-polar lipid fraction from 41.3±4.6% (phase II) to up to 7% (phase III and IV) (Fig 2D and 2E; S1 Table). Furthermore, oleic acid was observed as the most abundant fatty acid within the non-polar fraction during Phase II (linear growth phase) in accordance with previous studies [72], however their level decreased during further cultivation. Another noteworthy finding was the presence of very long chain fatty acids (VLCFA) such as hexacosenoate (C_26:1_), octacosenoate (C_28:1_), octacosadienoate (C_28:2_), tricontenoate (C_30:1_) in both polar and non-polar lipid fractions (Fig 2D and 2E), since these VLCFA were reported to originate from oleic acid and to function as precursor for rigid polymers associated with the structure of *B*. *braunii* outer cell walls [3]. Interestingly, the VLCFAs derived from the polar lipid fraction increased in their relative abundance, while they were found to be decreased in the non-polar fraction during cultivation (Fig 2D and 2E).

However, the non-polar lipid fraction of CCAP 807/2 appeared to be more differentiated in comparison to the polar fraction at different stages of growth (Phase II–IV) and also contained hydrocarbons as they were co-extracted (Fig 2E). While, all the fatty acids from the non-polar lipid fraction showed a similar response and decreased over the period of cultivation, the abundance of hydrocarbons within the same fraction increased dramatically over the time course of the cultivation (Fig 2E). The detected hydrocarbons in the derivatized non-polar lipid fraction of *B*. *braunii* CCAP 807/2 are composed of alkadienes such as C_23_H_44_, C_25_H_48_, C_27_H_52,_ C_29_H_56_ and C_31_H_60_ and of alkatrienes such as C_25_H_46_, C_27_H_50_, C_29_H_54_, and C_31_H_58_. Among these detected hydrocarbons, C_27_H_52_ and C_29_H_56_ were the most abundant hydrocarbons accounting to 4.7±2.0% and 14.9±5.3% in the linear phase (Phase II), and further increased to 11.6±1.6% and 41.7±7.3% during the stationary phase (Phase III), respectively (Fig 2E, ii; S1 Table).

The comparison of the relative levels of hydrocarbons and fatty acids derived from the total non-polar lipids fraction (considering the total non-polar lipids at Phase II as 100%) revealed that the hydrocarbons increased from approximately 27% during Phase II (linear phase) to up to 81% during Phase III (stationary phase), with a further substantial increase to up to 132% at the end of cultivation (Phase IV) (Fig 2F). Hence, these data illustrate that hydrocarbons represent the major components of the non-polar lipid fraction of *B*. *braunii* CCAP 807/2, in contrast to other *B*. *braunii* race A strains such as UTEX 2441 [17]. Notably, at the same time, a drastic decrease could be observed in the abundance of oleic acid (C_18:1n9c_) from 41.3±4.6% (Phase II) to 7.7±2.8% (Phase III) within the non-polar lipid fraction (Fig 2E, S1 Table). Oleic acid was reported to be markedly predominant with more than 80% of total fatty acid in *B*. *braunii* race A [72], and this very high level, and the similarity structure and stereochemistry of alkadienes suggest that oleic acid is the direct precursor of the *n*-alkadienes and *n*-alkatrienes [3,11,20]. Furthermore, the intracellular concentration of oleic acid was reported to remain low during the elevated production of hydrocarbons; however, when hydrocarbon production declines, the oleic acid concentration rises sharply [11]. Therefore, the results obtained in the present work concerning the precursor-product relation (oleic acid level remains low during hydrocarbon production) correlate well with earlier studies. However, contrary to the previous observations of a high hydrocarbon production during the exponential and early linear growth phase [11,13,20], the intracellular metabolic analysis of *B*. *braunii* CCAP807/2 reveals a reduction of the oleic acid concentration and a simultaneous increase in the hydrocarbon abundance to occur mainly during Phase III (Fig 2E). Phase III was assessed to represent the stationary growth phase, based on results obtained within the present study such as chlorophyll and protein content, C/N ratio as well as nitrate concentration in the culture supernatant (Fig 1B–1D; S1 Fig), most likely triggered by nutrient depletion of the culture medium. Furthermore, the analyses of the metabolite as well as pigment profiles (Fig 2A and 2B) reaffirm this conclusion.

### 3.3. Extracellular product formation

In addition to the investigation of the intracellular metabolic response during the proposed growth phases (Fig 2), easily accessible products were analyzed, such as liquid hydrocarbons, which are secreted by the *B*. *braunii* cells into the extracellular matrix [18,24] and carbohydrates (polysaccharides) that are accumulating in the culture medium supernatant [3,8].

**Fig 3:**
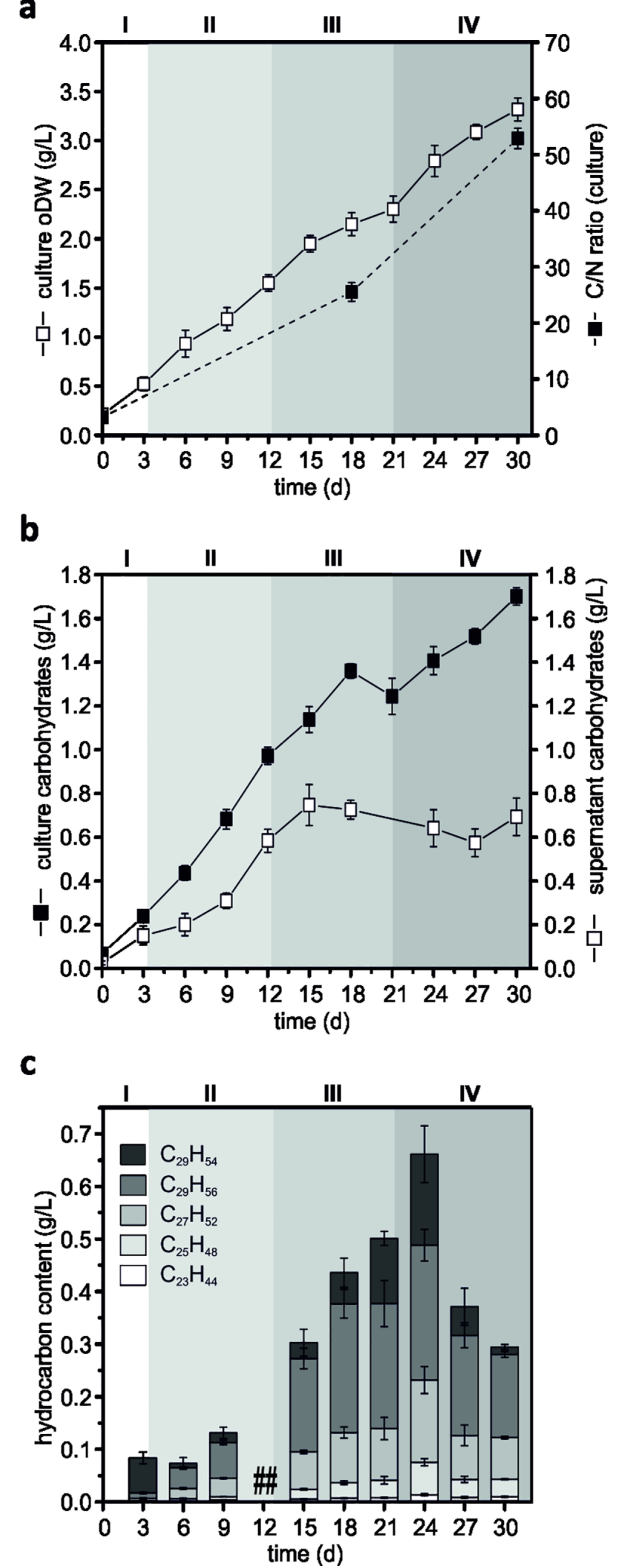
Extracellular product formation of *Botryococcus braunii* CCAP 807/2 in form of carbohydrates and hydrocarbons during the proposed growth stages referred as Phases I (lag phase), II (linear phase), III (stationary phase) and IV (decline phase). **a.** Determination of organic dry weight (oDW) and C/N ratio of the whole culture broth (containing cells and supernatant) over the period of cultivation for 30 days. **b.** Determination of total carbohydrate concentration in the whole culture broth and the cell-free supernatant. **c.** Quantification of total extractable hydrocarbons via GC-FID at each time point during cultivation (except for day 12 –lost samples (##)). Error bars represent standard error (SE; n = 9 for **a**, n = 12 for **b**) and standard deviation (SD) for **c**.

The determination of the total organic dry biomass weight (oDW) of the whole culture broth (including cells and culture supernatant) was performed for the product formation analysis, since previous studies with *B*. *braunii* revealed that the viscosity of the culture increases with the progress of the cultivation due to exopolysaccharide accumulation [19,23]. In contrast to the cell oDW (Fig 1B), the analysis of the whole culture broth showed an overall higher biomass content (with up to 0.7 g L^-1^ depending on the sample time point). The biomass yield accounted 3.3±0.1 g L^-1^ at the end of cultivation (Phase IV), with a maximum biomass productivity of up to 118 mg L^-1^ d^-1^ (Fig 3A). Accordingly, also the C/N ratio of the whole culture broth was measured, revealing a similar behavior as observed before for the total cell biomass (Fig 1B, C/N ratio of 43.9±1.9 at the end of the cultivation), with even higher ratios of 52.9±0.3 (Fig 3A) due to accumulation of carbon-rich compounds in the culture supernatant. Thereby, this observation also supports the proposition of distinct growth phases and ongoing nutrient limitation of the *B*. *braunii* CCAP 807/2 cultures.

Owing to the fact that *B*. *braunii* race A strains were reported for their capability to produce both hydrocarbons and (exo-)polysaccharides [3,19,27], the total amount of carbohydrates (of whole culture broth and cell-free culture supernatant) and *n*-hexane-extractable hydrocarbons from a *B*. *braunii* CCAP807/2 culture broth was determined at each harvesting time point (Fig 3). As a result, the amount of total carbohydrates for *B*. *braunii* CCAP 807/2 in the whole culture broth was shown to increase continuously during the different stages of the cultivation to a maximum of 1.7±0.2 g L^-1^ (Fig 3B, black squares), thus representing up to 52% of total culture oDW at the end of the culturing period of 30 days (Table 1). However, the maximum total carbohydrate content with respect to the organic dry weight was achieved between the Phases II and III (days 12–18, linear and stationary growth phases) with up to 63% of culture oDW and decreased significantly towards the end of the cultivation (Table 1). Thereby, the observed maximal carbohydrate content was in the upper range in comparison with other investigated *B*. *braunii* strains [27]. Thus, the *B*. *braunii* strain CCAP 807/2 may be considered as a high-yield carbohydrate producer.

**Table 1:** Overview of the whole culture biomass composition of *B*. *braunii* CCAP807/2 during the proposed growth stages.

	time	protein	carbohydrates		hydrocarbons
			total	external	
	(d)	% of culture oDW	% of culture oDW	% of culture oDW	% of culture oDW
**Phase I**	0	**23.83 ± 5.95**	31.75 ± 5.61	12.01 ± 4.32	nd
** **	3	**24.94 ± 4.43**	45.73 ± 3.57	28.71 ± 3.58	16.02 ± 2.72
**Phase II**	6	**23.12 ± 4.40**	46.65 ± 2.44	21.44 ± 1.25	7.88 ± 1.68
** **	9	**22.95 ± 2.70**	**57.75 ± 1.13**	26.12 ± 2.21	10.92 ± 1.38
** **	12	18.89 ± 5.16	**62.7 ± 1.76**	**37.73 ± 0.60**	nd
**Phase III**	15	16.35 ± 1.38	**58.34 ± 3.03**	**38.31 ± 4.81**	15.53 ± 2.58
** **	18	15.16 ± 3.04	**63.31 ± 1.53**	**33.79 ± 2.00**	**20.3 ± 3.25**
** **	21	15.51 ± 3.86	54.04 ± 3.59	nd	**21.77 ± 3.77**
**Phase IV**	24	13.18 ± 2.05	50.38 ± 2.30	22.97 ± 3.02	**23.68 ± 4.25**
** **	27	12.54 ± 1.59	49.19 ± 1.13	18.6 ± 2.06	11.99 ± 2.81
** **	30	11.2 ± 1.38	51.28 ± 0.74	20.9 ± 2.59	8.81 ± 0.45

The growth stages are referred as Phases I (lag phase), II (linear phase), III (stationary phase) and IV (decline phase). Shown are the values of total proteins and carbohydrates (including the proportion of the external carbohydrates) as well as the n-hexane extractable hydrocarbons, with maximum values marked in bold. oDW: organic dry weight; nd: not determined

The measurement of total external carbohydrates from the cell-free supernatant revealed a continuous increase till day 15 to up to 0.78 g L^-1^, i.e. during the Phases II and III (linear and stationary phase, respectively) and stagnated thereafter (Fig 3B, white squares). Thus, similarly to the accumulation behavior of the total carbohydrates, the active accumulation of the external carbohydrates of up to 38% of culture oDW could be observed between the Phases II and III (i.e. during linear and stationary growth phases), while no further enhancement was observed in later growth phases (Table 1). Possible explanations for this observation could be the occurrence of the detachment of carbohydrates (excised segments of the algal retaining cell wall and its fibril sheath) only during the active cell division of *B*. *braunii* [8] and/or the release of carbohydrate-hydrolyzing enzymes into the culture medium after cell lysis [73]. Based on the results of the physiological properties and intracellular metabolites of *B*. *braunii* CCAP807/2 obtained within the present study, the onset of the stationary growth phase occurred between the days 12–15, when the active cell division was diminished mainly due to the nutrient depletion of the culture medium (Figs 1 and 2; sections 3.1 and 3.2). Additionally, the presence of the bacterial consortium, co-existing with all *B*. *braunii* cells, might also play a role in the reduction of extracellular carbohydrates in the culture supernatant [3]. In addition, a detailed analysis of these cell-free supernatant carbohydrates revealed that besides the presence of rhamnose and uronate, galactose was the main component. The relative abundance of all these compounds gradually increased at later stages of cultivation, when compared to the linear growth phase (Phase II, day 6) (S3 Fig). This observation is in good agreement with earlier findings that galactose represents the major component of all heterogeneous polysaccharides produced by *B*. *braunii* [3,4,74] and may represent the polysaccharide chain backbone of the cell shells together with other saccharides as side chains [8]. Interestingly, the detection of raffinose as well as of the raffinose precursors sucrose, galactose, galactinol and myo-inositol (Fig 2A, i; section 3.2) implicate that the cell wall backbones of *B*. *braunii* consist of high molecular weight oligomers/polymers of the RFO family (raffinose family of oligosaccharides), comparable to higher plants [51]. However, this hypothesis may represent the subject of future studies and should be thoroughly investigated.

The evaluation of the total extracellular hydrocarbon content of CCAP807/2, extracted from the whole culture broth increased gradually, starting from day 9, and reached a maximal productivity of up to 40 mg L^-1^ d^-1^ between the days 15 and 24 (Phase III and start of Phase IV, proposed stationary and decline growth phases, respectively). Upon quantification, the qualitative analysis revealed C_29_H_56_ (nonacosadiene) to be the most abundant hydrocarbon followed by C_27_H_52_ (heptacosadiene) (S2 Table). Interestingly, on day 24 of the cultivation, the alkadienes C_29_H_56_ and C_27_H_52_ as well as the alkatriene C_29_H_54_ (nonacosatriene) were found to be the most prominent components. Other hydrocarbons such as C_25_H_48_ (pentacosadiene) and C_23_H_44_ (tricosadiene) as well as unidentified compounds were also detected in trace amounts (Fig 3C, S2 Table). Notably, the highest *n*-hexane-extractable hydrocarbon content with up to 0.66 g L^-1^ was measured on day 24 (start of Phase IV, alias growth decline phase), representing up to 24% of culture oDW and decreased massively afterwards towards the end of the cultivation (Fig 3C, Table 1). However, at the same time, the gravimetrically determined total lipid content remained constant (Fig 2C) and the non-polar fraction as well as the contaminant hydrocarbon amount increased during the time course of the cultivation (Fig 2E and 2F). This finding indicated that rather the *n*-hexane-extractable proportion of the hydrocarbons decreases and not the total hydrocarbon content of the culture. And indeed, Casadevall and co-workers suggested the reduction of hydrocarbons during prolonged stationary phase to be attributed to the aging of the culture, which might influence the preferential location of hydrocarbon accumulation [19].

Overall, *Botryococcus braunii* CCAP 807/2 cultures reached within the present study maximal production yields of up to 84% of oDW of the whole culture broth in form of carbohydrates and secreted hydrocarbons (Table 1). The observed findings clearly show that this strain possesses a higher carbon partitioning towards carbohydrates than lipids (particularly hydrocarbons), and are therefore consistent with previous assumptions that the low production of hydrocarbons contrasts with the high production yields of carbohydrates [23]. The attained values are comparatively high compared to the obtained yields of Gouveia and colleagues for the same strain, as they either achieved a high product concentration (up to 50% of DW) at low biomass productivity of 0.07 g L^-1^ d^-1^ or very low product yields (up to 12% of DW) at high biomass productivity of 0.14 g L^-1^ d^-1^ [27]. In addition, while the production of carbohydrates steadily increased from the beginning of the cultivation in agreement with previous studies [3,13,19], an enhanced hydrocarbon production was observed between the Phases III and IV (Table 1). The analysis of physiological parameter, which enabled the estimation of distinct physiological growth stages (Phase I–IV) of *B*. *braunii* CCAP807/2 (Fig 1, S1 Fig, section 3.1), revealed that the cultures entered nitrogen limitation from day 12 onwards followed by an increase in hydrocarbon production (Fig 3C, Phase III). Although, these results correspond well to the known behavior of green microalgae, meaning the high accumulation of fatty acids/triglycerides under nutrient limitation [21], they contradict previous reports of *B*. *braunii*, in which the highest hydrocarbon production was observed during active cell growth within the exponential and early linear growth phases [11,13,18,20]. It is certainly possible that the observed growth and product formation behavior of *B*. *braunii* CCAP807/2 is rather unique, but the results obtained within this work with simultaneous consideration of previous investigations [22,25,27] illustrate that the production efficiency of *Botryococcus* species varies greatly with the physiological state of the cells (strongly related to the availability of nutrients), showing production maxima for specific compounds (Table 1) at different growth phases.

## Conclusions

Collectively, the present study focused on the detailed characterization of *Botryococcus braunii* Race A strain CCAP 807/2, including the systematic investigation on growth, product formation behavior and associated metabolic responses.

As a result, the analyses of the intracellular metabolites in conjunction with the determined physiological parameters provided a clear picture of differentiated growth phases along with associated changes in the biomass composition. Depending on the particular specific growth phase, the biomass composition varied in its maximum proportions of the respective components. This first of its kind sequential metabolome analysis of a *B*. *braunii* strain revealed the appearance of compound-specific precursors during the respective growth phase. The observation of a constant expression of raffinose under standard conditions in a green microalga together with the observed high levels of sugar metabolites indicated a preferred carbon channeling towards carbohydrate products rather than to lipids in the strain CCAP 807/2.

Therefore, the comprehensive analysis performed here, provides a better understanding of the overall cell physiology of *B*. *braunii* and offers valuable insights into the productivity behavior, thus providing the necessary tools to identify the best time periods during cultivation for the production of specific compounds. Hence, the acquired data may also be vital for future ‘omics studies, such as transcriptomics and/or proteomics. Therefore, this study is a very good example that for industrial applications with *B*. *braunii* it is indispensable to systematically investigate in forehand the interconnection between growth behavior and product quantity/portfolio. These data are the pre-condition for suitable strain selection and for setting up the appropriate conditions for the efficient formation of products of interest.

## Supporting information

S1 Fig Protein concentration and pH measurements of the whole culture broth. The protein concentration was determined of the whole culture broth cell extracts at each time-point during the cultivation. Error bars represent standard error of mean value of three biological and four technical replicates (SE; n = 12). pH measurement of the cultures were taken periodically during the culturing and the error bars represent standard deviation of mean values of three biological and three technical replicates.
(TIF)

S2 Fig Total number and relative peak area abundance of the detected compounds in non-targeted metabolome analysis of *B*. *braunii* CCAP 807/2 during the proposed growth stages (Phases II–IV) by using GC-MS. Metabolites were identified by comparison with the NIST 05 library, the Golm Metabolome Database (Lib) and additionally verified with purified standards (Std). The unidentified metabolites with RSI values below 750 were considered as not identified (NI) as well as peaks with none information available from above mentioned databases (NA), were still included into total number of detected peaks.
(TIF)

S3 Fig Detailed GC-MS analysis of the external carbohydrates from the cell-free medium supernatant of *B*. *braunii* CCAP 807/2 cultures. **a.** GC-MS chromatogram after methanolysis and peracetylation of cell-free Chu media and culture supernatant during the proposed growth phases of *B*. *braunii*, represent by Phase II (linear phase at day 6), Phase III (stationary phase at day 15) and Phase IV (late stationary/decline phase at day 30). The numbers in the chromatogram represent 1). rhamnose, 2). uronate and 3). galactose, respectively. **b.** GC-MS chromatogram of spiking of samples with glucose and galactose to confirm the presence of galactose in the supernatant media.
(TIF)

S1 Table Listing of all detected metabolites of *B*. *braunii* CCAP 807/2, obtained during the present study by using different extraction methods and instruments (indicated in column C). Mean values from three biological and three technical replicates as well as standard deviation (SD) of all the metabolites detected during the proposed growth stages (Phases II–IV) are shown in columns E–J. The fold change (up or down) of metabolites during Phases III and IV with respect to Phase II are shown in columns K–AH).
(XLSX)

S2 Table Listing of all detected metabolites within the *n*-hexane extractable fraction. Listing of all peaks and their relative abundance level detected in the *n*-hexane extractable fraction via GC-FID (columns B–O) for each cultivation time point during the cultivation (except day 12 because of sample loss), including all identified and unidentified compounds. Additionally, columns R–AF show the relative abundance level of the identified peaks, based on the obtained mass spectra and available literature [10,17], detected in the *n*-hexane extractable fraction via GC-MS.
(XLSX)
