# Supplementary figures and images for: Metabolic survey of Botryococcus braunii: Impact of the physiological state on product formation
Source: PLoS One. 2018 Jun 7;13(6):e0198976. doi: 10.1371/journal.pone.0198976 (PMC5991718; doi:10.1371/journal.pone.0198976)

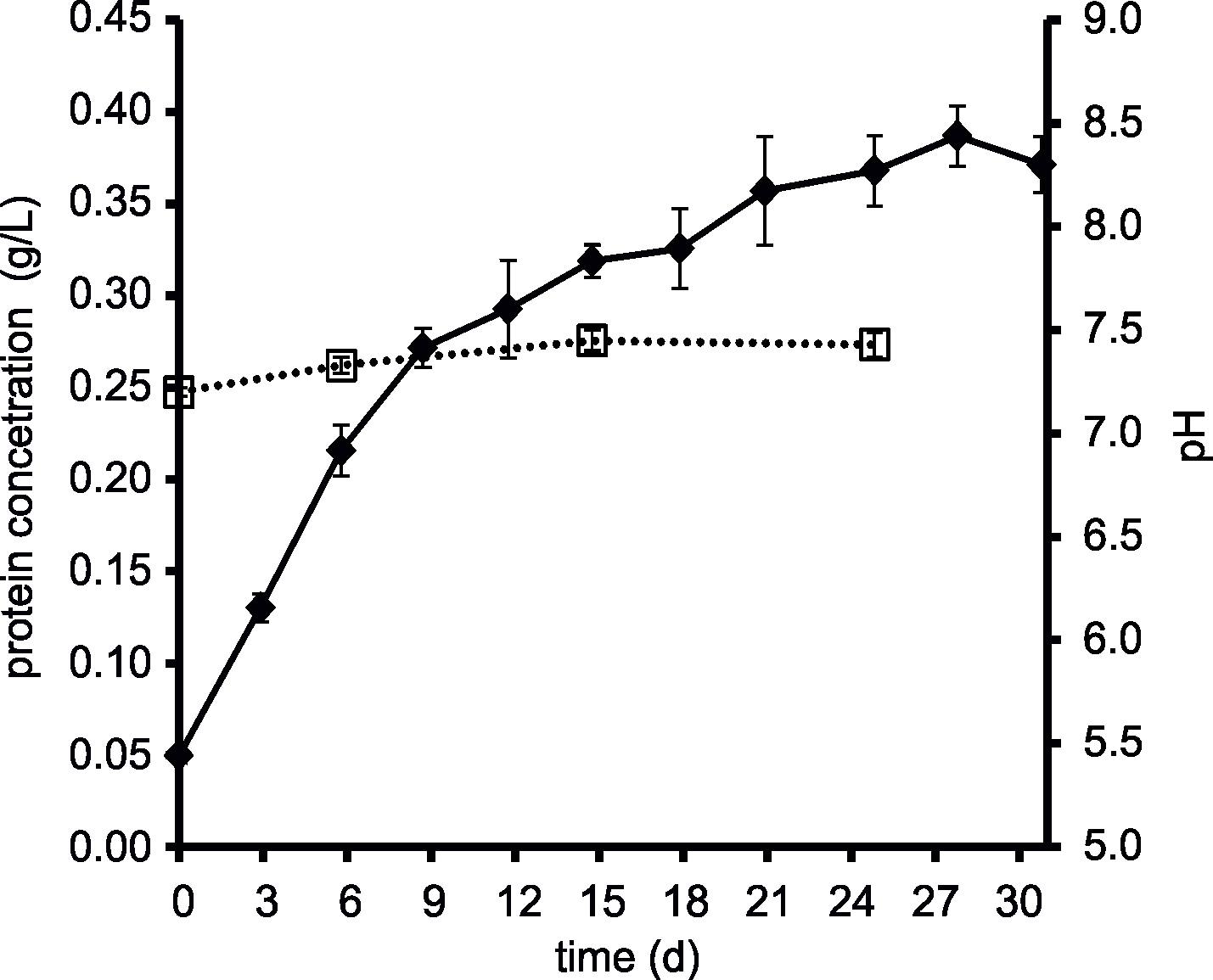

Supplement: S1 Fig — The protein concentration was determined of the whole culture broth cell extracts at each time-point during the cultivation. Error bars represent standard error of mean value of three biological and four technical replicates (SE; n = 12). pH measurement of the cultures were taken periodically during the culturing and the error bars represent standard deviation of mean values of three biological and three technical replicates. (TIF) [file pone.0198976.s001.tif]

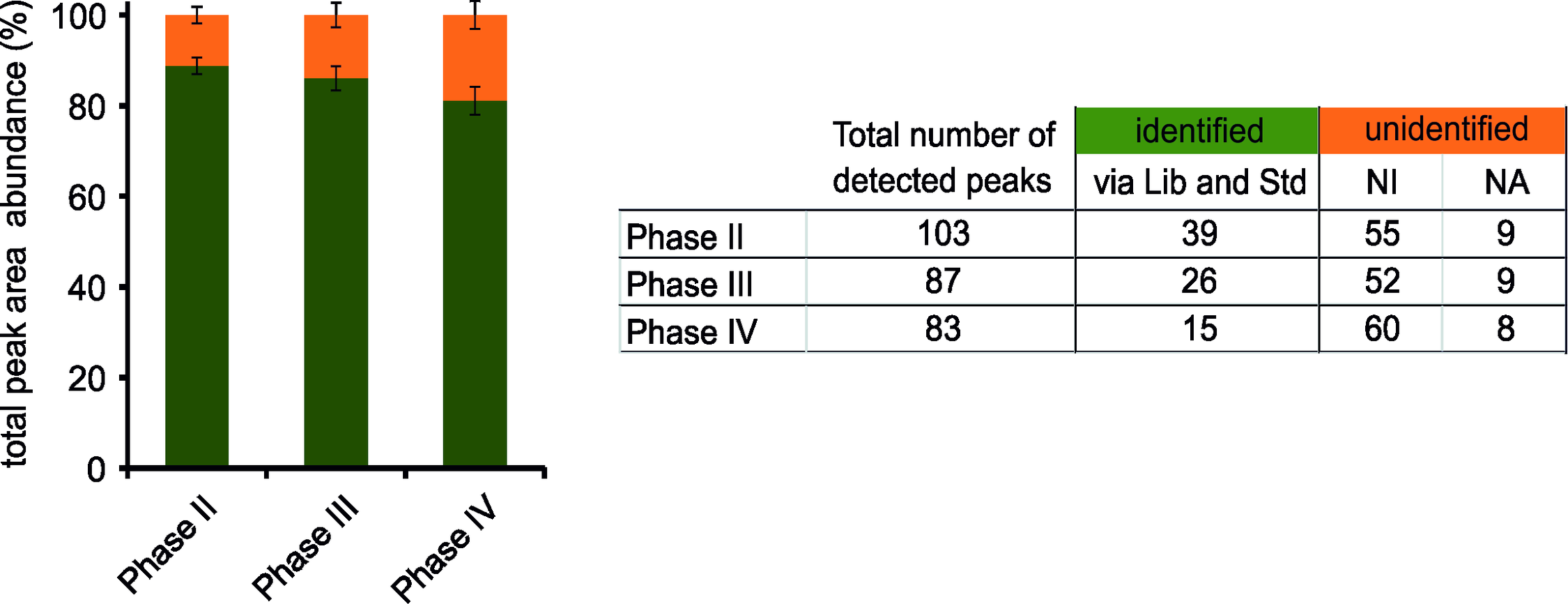

Supplement: S2 Fig — Metabolites were identified by comparison with the NIST 05 library, the Golm Metabolome Database (Lib) and additionally verified with purified standards (Std). The unidentified metabolites with RSI values below 750 were considered as not identified (NI) as well as peaks with none information available from above mentioned databases (NA), were still included into total number of detected peaks. (TIF) [file pone.0198976.s002.tif]

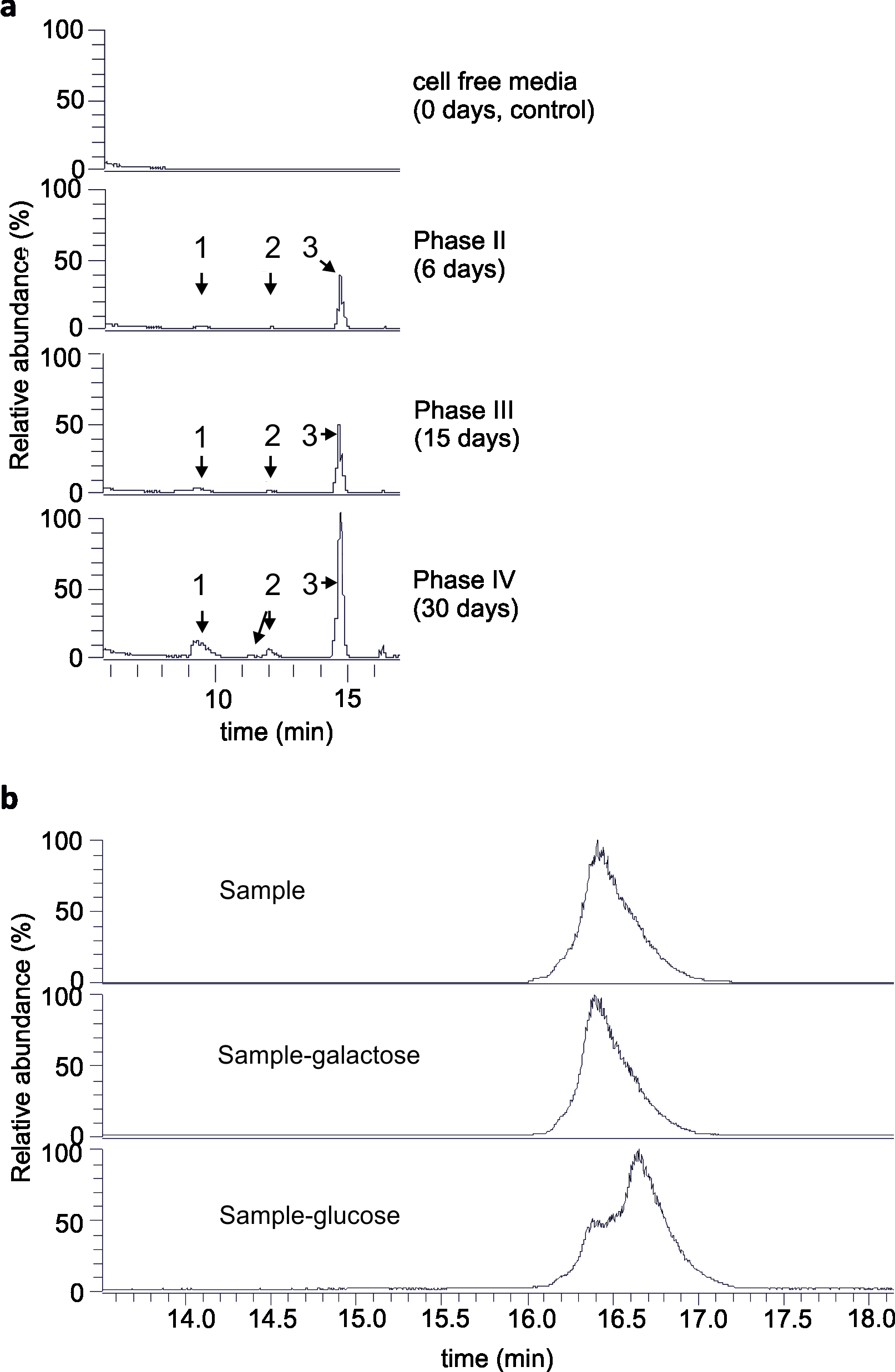

Supplement: S3 Fig — a. GC-MS chromatogram after methanolysis and peracetylation of cell-free Chu media and culture supernatant during the proposed growth phases of B. braunii, represent by Phase II (linear phase at day 6), Phase III (stationary phase at day 15) and Phase IV (late stationary/decline phase at day 30). The numbers in the chromatogram represent 1). rhamnose, 2). uronate and 3). galactose, respectively. b. GC-MS chromatogram of spiking of samples with glucose and galactose to confirm the presence of galactose in the supernatant media. (TIF) [file pone.0198976.s003.tif]
